# The Dectin-1 Receptor Signaling Pathway Mediates the Remyelination Effect of Lentinan through Suppression of Neuroinflammation and Conversion of Microglia

**DOI:** 10.1155/2022/3002304

**Published:** 2022-12-28

**Authors:** Dandan Zhang, Yue Jia, Xingzong Sun, Haoran Li, Min Yin, Hongliang Li, Lili Dai, Li Han, Lei Wang, Menghan Qian, Jing Du, Keming Zhu, Hongkun Bao

**Affiliations:** ^1^School of Medicine, Yunnan University, Kunming 650091, China; ^2^Department of Gynecology, The Third Affiliated Hospital of Kunming Medical University (Yunnan Cancer Hospital), Kunming 650118, China; ^3^School of Agronomy and Life Sciences, Kunming University, Kunming 650214, China; ^4^The National Clinical Research Center for Mental Disorders & Beijing Key Laboratory of Mental Disorders, Beijing Anding Hospital & Advanced Innovation Center for Human Brain Protection, Capital Medical University, Beijing 100088, China

## Abstract

Demyelinating diseases such as multiple sclerosis (MS) are chronic inflammatory autoimmune diseases and involve demyelination and axonal degeneration. Microglia rapidly respond to changes in the environment by altering morphotype and function during the progressive disease stage. Although substantial progress has been made in the drug development for MS, treatment of the progressive forms of the disease remains unsatisfactory. There is great interest in identifying novel agents for treating MS. *Lentinus edodes* is a traditional food, which can improve physiological function. Lentinan (LNT), a type of polysaccharide extracted from mushroom *Lentinus edodes*, is an anti-inflammatory and immunomodulatory agent. Here, we studied the remyelination effects of LNT and its therapeutic target in regulating the functions of neuroinflammation. We found that LNT enhanced remyelination and rescued motor deficiency by regulating dectin-1 receptor to inhibit neuroinflammation and microglial cell transformation. LNT promoted the conversion of microglial cells from the M1 status induced by LPS to the M2 status, enhanced the anti-inflammatory markers IL-10 and BDNF, inhibited inflammatory markers TNF-*α* and IL-1*β*, and downregulated the microglia activation and oligodendrocyte and astrocyte proliferation by modulating dectin-1. If we injected the dectin-1-specific inhibitor laminarin (Lam), the remyelination effects induced by LNT were completely abolished. Thus, these results suggest that LNT is a novel and potential therapeutic agent that can rescue MS neuroimmune imbalance and remyelination through a dectin-1 receptor-dependent mechanism.

## 1. Introduction

Inflammatory demyelinating disease of the central nervous system (CNS) is a disease that involves multiple parts of the brain and spinal cord [1]. Currently, there is no efficient drug to cure this disease completely. The development of new drugs to treat demyelinating diseases by “promoting myelin regeneration” will bring hope for the complete cure of such diseases. Repair of inflammatory demyelinating lesions in MS requires microglia to clear myelin debris and switch from a proinflammatory to an anti-inflammatory lesion environment. Microglia are resident macrophages of the CNS [2] and have important physiological functions in maintaining tissue homeostasis and contribute to CNS pathology. Evidence has shown that microglia play a key role in both active inflammation and remyelination [3]. Microglia rapidly respond to changes in the environment by altering morphotype and function, with both neuroinflammatory and neuroprotective properties, illustrating the plasticity of these cells [4].

*Lentinus edodes* is a kind of high protein and low fat food with rich nutrition and high medicinal value. LNT, a type of polysaccharide extracted from mushroom *Lentinus edodes*, is the most important medicinal physiological active component of *Lentinus edodes*, which has significant effects on regulating body immunity, antitumor, antivirus, anti-inflammation, and antioxidation [5]. This mushroom polysaccharide has no harm and places few side effects on the human body [6]. LNT is a *β*-(1→3)-D-glucan, and it is known to be a type of biologically active macromolecule. Cumulative studies have shown that it has strong anti-inflammatory and immunomodulatory functions [7–10]. Dectin-1 specifically recognizes *β*-(1→3)-linked glucans and is an immune regulator in the CNS [11]. Previous studies have found that *β*-glucan can promote regeneration and repair after optic nerve system injury through dectin-1 receptors [12]. Dectin-1 is one of the best-characterized C-type lectin receptors in mice and in humans [13]. It is predominantly expressed on myeloid cells, including monocytes, macrophages, dendritic cells, and neutrophils [14]. In addition, the dectin-1 receptor is also found in microglial cells in the CNS [15], suggesting its role in regulating the neuroimmune system in the brain. However, it is not clear whether LNT regulates microglia through dectin-1 receptors to inhibit inflammation-mediated remyelination. In this study, we systematically investigated the effect of LNT on remyelination and microglial regulation through dectin-1 receptors both in vitro and in vivo.

## 2. Materials and Methods

### 2.1. The BV2 Cell Culture

BV2 cells were cultured in DMEM high glucose complete medium supplemented with 10% fetal bovine serum (FBS) (Cat.: A3160802, Gibco) and 1% penicillin streptomycin solution at 37°C in a humidified atmosphere of 5% CO_2_ in T25 flasks or T75 flasks. When the cells reached over 80% confluence, they were seeded into 96-well, 24-well, or 6-well plates for further experiments.

### 2.2. Cell Counting Kit-8 (CCK-8) Assay

To determine cell viability, a CCK-8 assay was applied in this study. BV2 cells (2 × 10^3^ cells/well) were plated in 96-well plates, and all of the experiments were conducted 24 h after the cells were seeded. Cells were then treated with lipopolysaccharide (LPS) (Cat.: L2630, Sigma), LNT (Cat.: SL8730, Solarbio), and laminarin (Lam) (Cat.: L9634, Sigma) for 24 h. Cell viability was determined by CCK-8 assay following the manufacturer's instructions (Cat.: BS350A, Biosharp).

### 2.3. Cell Drug Treatments

BV2 cells were seeded in 6-well plates at a density of 1 × 10^6^ cells per well and incubated overnight. Cells were stimulated with LPS (1 *μ*g/ml) as an inflammatory state in vitro. To study the effect of LNT on LPS-induced neuroinflammation, LNT was administered simultaneously with LPS to BV2 cells. After 24 h of treatment, the assay was performed following the methods provided. For the study of Lam (a selective dectin-1 receptor antagonist) on LNT and LPS-cotreated BV2 cells, Lam was administered to BV2 cells 2 h before LPS and LNT treatment.

### 2.4. Animals

Male C57BL/6 mice (6-7 weeks old; weighing 20 ± 2 g) were purchased from Vital River Laboratories (Beijing, China). The animals were maintained under a 12 h light/dark cycle with access to food and tap water pellets *ad libitum*. The mice were housed in the Yunnan University and performed in accordance with the international guidelines. Animal protocols were approved by the Yunnan University Institutional Animal Care and Use Committee.

### 2.5. Cuprizone (CPZ) Administration and Drug Treatment

The demyelination was induced by adding 0.2% CPZ (Cat.: 14690, Sigma) to standard rodent chow. Mice were fed with chow containing CPZ for 5 weeks. Control groups received a normal diet without cuprizone. Food was monitored daily. The mice were given drug treatment after CPZ withdrawal for 1 week. LNT and Lam were dissolved in 0.9% saline. LNT was intraperitoneally administered at doses of 4 mg/kg, 10 mg/kg, and 20 mg/kg once daily for 1 week during the remyelination period, and Lam was intraperitoneally administered at a dose of 10 mg/kg 2 h before LNT injection.

### 2.6. Rotarod Test

The rotarod test was conducted as previously described [16]. The rotarod test was used to evaluate the motor coordination of the mice at the end of the experimental procedure. Each mouse was trained on the cylinder at 10 rpm for 300 s and repeated 3 days before the test. The purpose of the training was to improve the animals' skill and avoid fortuitous falling. All mice were tested on the rod at 30 rpm for 180 s at the end of the experimental period, and the time required for a mouse to fall off from the rotating rod to the floor was recorded. All mice were tested on the rotarod three times. The average latency was calculated from three measurements.

### 2.7. Brain Tissue Collection

Mice were deeply anesthetized by intraperitoneal injection of diethyl ether (Cat.: 60-29-7, Chron Chemicals) and perfused with PBS and 4% paraformaldehyde (PFA). Brains were removed after perfusion and postfixed in the same fixative at 4°C overnight and then dehydrated in gradient sucrose phosphate buffer in sequence. Coronal sections (20 *μ*m) were cut at the fornix region of the corpus callosum for analysis and stored at -80°C.

### 2.8. Luxol Fast Blue (LFB) Staining

To assess myelin loss and the degree of demyelination of the corpus callosum, brain sections were stained with LFB solution. For staining, sections were first dehydrated in 70%-95% gradient ethanol and then incubated with 0.1% LFB solution for 40-50 min at 60°C. Sections were further incubated in 0.5% lithium carbonate solution, followed by differentiation in ethanol solution. Finally, the brain sections were sealed with neutral gum (Cat.: G8590, Solarbio).

### 2.9. Western Blotting

Total proteins of the corpus callosum were extracted using RIPA lysis buffer, and their concentration was measured using a BCA kit (Cat.: 23227, Thermo Scientific). Proteins were separated by 10-12.5% SDS-polyacrylamide gels and transferred onto PVDF membranes after electrophoresis. The membranes were incubated with 1% BSA for 1 h and the primary antibodies overnight at 4°C. After the membrane was incubated with the corresponding secondary antibodies for 1 h at room temperature, immunolabeled proteins were detected and visualized using the ECL substrate (Cat.: BL520B, Biosharp). The band was developed with an enhanced chemiluminescence system (Amersham Imager 600, GE), and the band intensity was analyzed using Image-Pro Plus version 6.0 software.

The primary antibodies used in this research were as follows: anti-Iba1 (Cat.: AB5076, Abcam, 1 : 1000), anti-iNOS (Cat.: AB15323, Abcam, 1 : 1000), anti-Arg-1 (Cat.: AB91279, Abcam, 1 : 1000), anti-IL-1*β* (Cat.: AF-401-NA, R&D,1 : 1000), anti-IL-10 (Cat.: AB189392, Abcam, 1 : 1000), anti-TNF-*α* (Cat.: AF-410-NA, R&D,1 : 1000), anti-BDNF (Cat.: AB108319, Abcam, 1 : 1000), and anti-dectin-1 (Cat.: AB140039, Abcam, 1 : 500). The secondary antibodies were donkey anti-goat IgG (H+L) HRP (Cat.: A15999, Invitrogen,1 : 5000), goat anti-rabbit IgG (H+L) HRP (Cat.: S0001, Affinity,1 : 5000), and goat anti-mouse IgG (H+L) HRP (Cat.: S0002, Affinity,1 : 5000). Anti-GAPDH (Cat.: AB8245, Abcam, 1 : 5000) and anti-beta tubulin (Cat.: MA5-11732, Thermo Scientific, 1 : 5000) were applied for loading calibration.

### 2.10. Immunofluorescence Staining

BV2 cells were seeded on 12 mm glass coverslips in 24-well plates overnight. After drug treatments, the cells were fixed with 4% paraformaldehyde and permeabilized using PBST. Then, the cells were blocked with 1% BSA for 1 h and incubated with primary antibodies overnight. After incubation, the cells were washed with PBST to remove the excess primary antibodies and incubated with fluorescent secondary antibodies. After that, the cells were mounted onto glass slides with DAPI Fluoromount-G (Cat.: 36308ES11, Yeasen Biotech Co., Ltd.).

Brain sections were washed in medium PBST for 15 min and incubated with 1% BSA for 1 h. Next, brain sections were incubated with primary antibodies and secondary antibody solution. After that, brain sections were mounted onto the slides with DAPI Fluoromount-G. The primary antibodies used in this research were as follows: anti-Iba1 (Cat.: AB1532, Abcam, 1 : 200), anti-iNOS (Cat.: AB15323, Abcam, 1 : 500), anti-Arg-1 (Cat.: AB91279, Abcam, 1 : 500), anti-dectin-1 (Cat.: A15999, Invitrogen,1 : 200), anti-MBP (Cat.: AB40390, Abcam, 1 : 200), anti-Olig2 (Cat.: AB109186, Abcam, 1 : 200), and anti-GFAP (Cat.: 16825-1-AP, Proteintech, 1 : 200). The secondary antibodies included Alexa Fluor 488-labeled donkey anti-goat (Cat.: 805-545-180, Jackson, 1 : 500), Alexa Fluor 594-labeled donkey anti-rabbit (Cat.: 711-585-152, Jackson, 1 : 500), and Alexa Fluor 488-labeled donkey anti-mouse (Cat.: 715-545-150, Jackson, 1 : 500).

### 2.11. Statistical Analysis

All data are presented as the mean ± SEM. Quantitative data were analyzed and graphed using GraphPad Prism 5 (GraphPad Software, Inc., San Diego, CA). All data were analyzed with one-way ANOVA followed by Tukey's post hoc test. *P* values < 0.05 were considered statistically significant.

## 3. Results

### 3.1. LNT Inhibited LPS-Induced BV2 Cell Activation In Vitro

**Figure 1:**
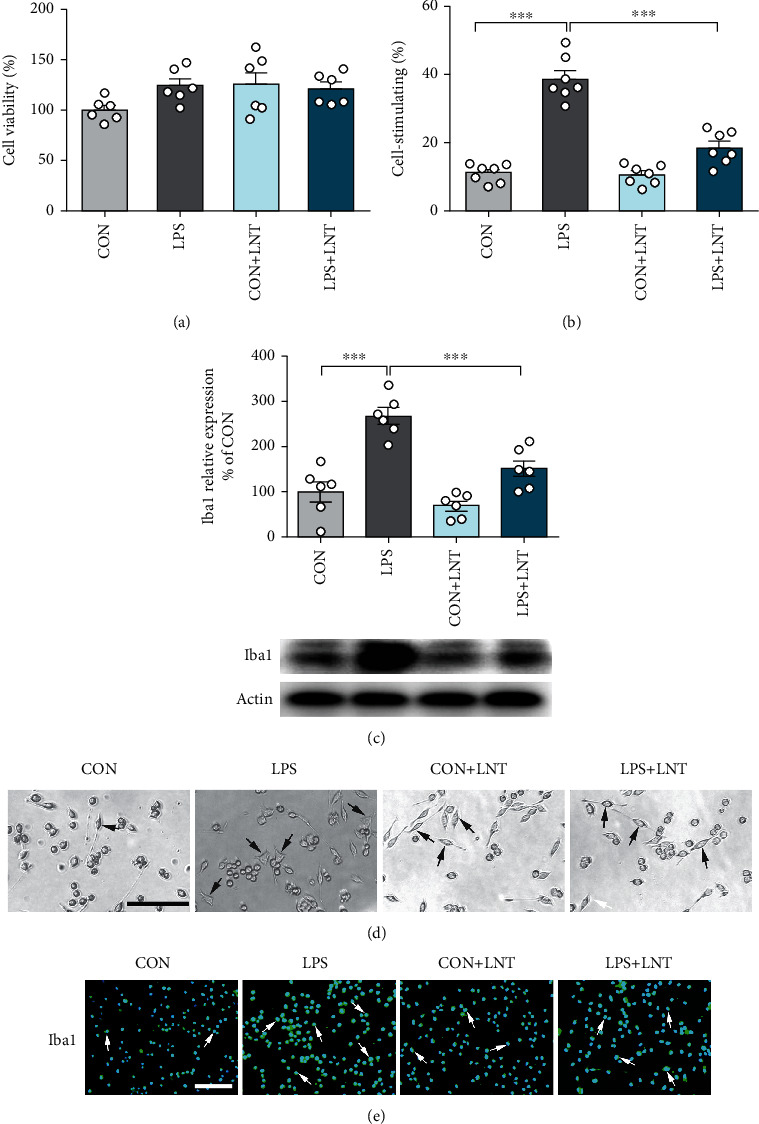
LNT inhibited LPS-induced activation of BV2 cells. The proliferation rate (percentage of control) of BV2 cells was measured by CCK-8 assay. (a) BV2 cells were treated with LNT (400 *μ*g/ml), LPS (1 *μ*g/ml), and LNT (400 *μ*g/ml) with LPS (400 *μ*g/ml) for 24 h. (b) The graph represents the quantification of activated BV2 cells. (c) The protein levels of Iba1 were detected by Western blotting. (d) Typical morphological features of cells were detected by microscopy. (e) Representative immunostaining showing the distribution of Iba1 in the experimental groups. Black or white arrows indicate representative cells in the treatment group. All data are presented as the mean ± SEM. Scale bar = 50 *μ*m (d); scale bar = 100 *μ*m (e). Statistical analysis was performed using one-way ANOVA followed by the post hoc Tukey tests. (^∗^*P* < 0.05, ^∗∗^*P* < 0.01, and ^∗∗∗^*P* < 0.001.)

To determine whether LNT inhibits LPS-induced BV2 cell activation, a CCK-8 assay was applied to determine the effect of LPS and LNT on the viability of BV2 cells. As shown in Figure 1(a), BV2 cells were cultured with LNT (400 *μ*g/ml), LPS (1 *μ*g/ml), and LNT (400 *μ*g/ml) with LPS (1 *μ*g/ml) for 24 h, and the results showed that LNT (400 *μ*g/ml) and LPS (1 *μ*g/ml) had no cytotoxic effect on BV2 cells. Finally, we selected doses of 400 *μ*g/ml LNT and 1 *μ*g/ml LPS for further study.

LNT suppressed LPS-induced BV2 cell activation. Activation of microglia is one of the prominent features of central nervous inflammation. Typical morphological features of cells were detected by microscopy (Figures 1(b) and 1(d)) and cellular immunofluorescence (Figure 1(e)). The morphology of control BV2 cells showed a small soma with distal arborization, a characteristic of “ramified” microglia. LPS-treated BV2 cells had shorter protrusions and enlarged and amoeba-shaped cell bodies. Compared with the control group, the percentage of cells being activated (38.46 ± 2.40%) (*P* < 0.001) and the expression of Iba1 were obviously upregulated, and LNT treatment significantly suppressed the percentage of cells being activated (18.43 ± 1.81%) (*P* < 0.001) and the expression of Iba1. The results were also confirmed by Western blotting (Figure 1(c)).

### 3.2. LNT Inhibited the LPS Stimulation-Induced Inflammatory Response in BV2 Cells

**Figure 2:**
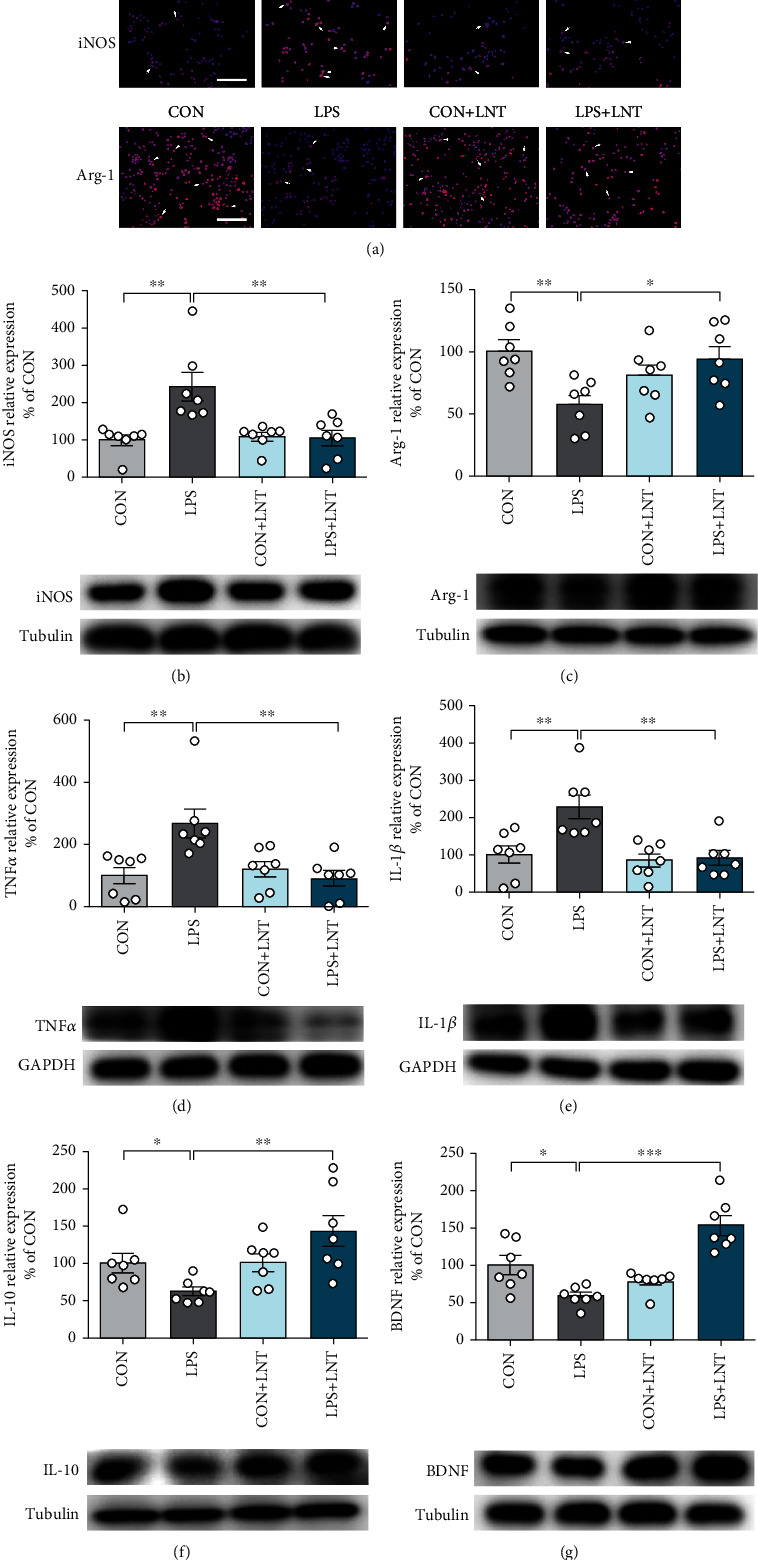
LNT inhibited LPS-induced microglial cell inflammation in BV2 cells. (a) Representative images showing the expression of iNOS and Arg-1 in the experimental groups. The protein levels of iNOS (b), Arg-1 (c), TNF-*α* (d), IL-1*β* (e), IL-10 (f), and BDNF (g) were detected by Western blot. White arrows indicate representative cells in the treatment group. All data are presented as the mean ± SEM. Scale bar = 50 *μ*m (a). Statistical analysis was performed using one-way ANOVA followed by the post hoc Tukey tests. (^∗^*P* < 0.05, ^∗∗^*P* < 0.01, and ^∗∗∗^*P* < 0.001.)

Lipopolysaccharide is regarded as a classic M1 microglial inducer that causes the M1 phenotype to express proinflammatory cytokines [17]. To evaluate M1/M2 polarization, we examined the expression of M1 and M2 cell markers (iNOS and Arg-1, respectively) using Western blotting. Compared with the control group, the expression of iNOS was greatly increased after LPS stimulation (*P* < 0.01). LNT suppressed the expression of iNOS in LPS-stimulated BV2 cells compared with LPS alone (*P* < 0.01) (Figure 2(b)). In contrast, the expression of Arg-1 was greatly decreased after LPS stimulation (*P* < 0.01), and LNT treatment significantly inhibited the downregulation of Arg-1 in LPS-induced BV2 cells (*P* < 0.05) (Figure 2(c)). LNT had little impact on microglial polarization in the resting state. Immunofluorescence images showed similar results (Figure 2(a)). In other words, LNT promoted the shift of M1 microglia to M2 microglia in LPS-treated BV2 cells.

To investigate the anti-inflammatory effects of LNT, the expression of inflammatory mediators was detected in this study by Western blotting, and we detected the levels of TNF-*α*, IL-1*β*, IL-10, and BDNF released into BV2 cells. The results showed that compared with the control group, the expression of TNF-*α* (*P* < 0.01) and IL-1*β* (*P* < 0.01) was significantly increased after LPS stimulation, and LNT downregulated the production of TNF-*α* (*P* < 0.01) and IL-1*β* (*P* < 0.01) induced by LPS (Figures 2(d) and 2(e)). However, the expression of IL-10 and BDNF was reversed in this study (Figures 2(f) and 2(g)). These results further confirmed that LNT can promote the conversion of BV2 cells from the M1 to the M2 phenotype after LPS stimulation.

### 3.3. The Dectin-1 Receptor Plays a Key Role in LNT-Inhibited Inflammatory Reactions

**Figure 3:**
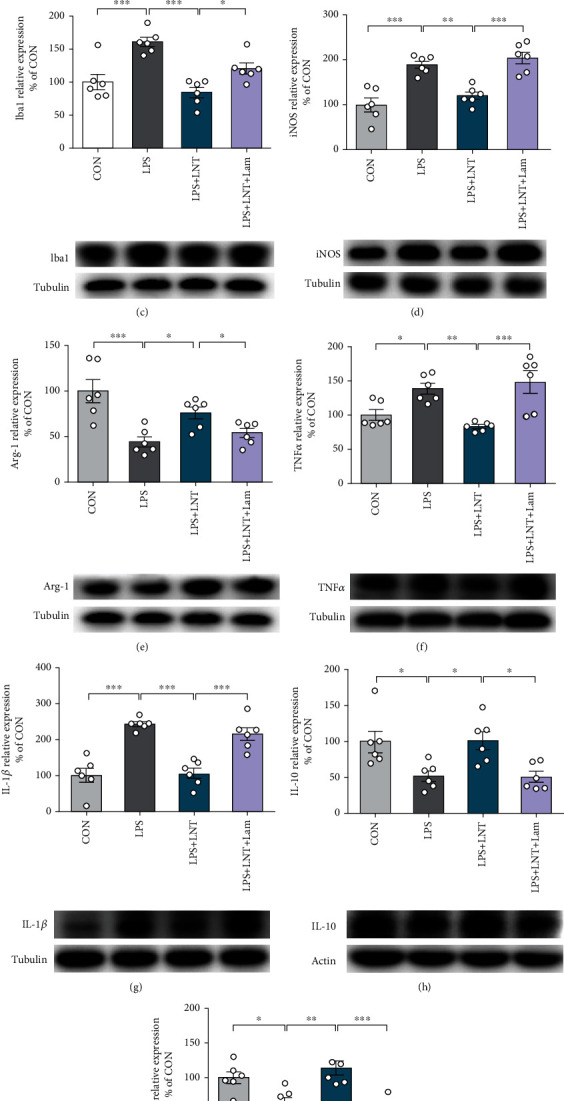
LNT, by modulating the dectin-1 receptor, inhibited LPS-induced microglial cell inflammation in BV2 cells. (a) The protein level of dectin-1 was detected by Western blot. (b) The survival rate of BV2 cells was measured by CCK-8 assay with different concentrations of Lam for 24 h. The protein levels of Iba1 (c), iNOS (d), Arg-1 (e), TNF-*α* (f), IL-1*β* (g), IL-10 (h), and BDNF (i) were detected by Western blot. All data are presented as the mean ± SEM. Statistical analysis was performed using one-way ANOVA followed by the post hoc Tukey tests. (^∗^*P* < 0.05, ^∗∗^*P* < 0.01, and ^∗∗∗^*P* < 0.001.)

Dectin-1 is a *β*-glucan (including LNT) receptor that regulates immune functions in many immune cell types [11, 13]. We examined dectin-1 expression in BV2 cells of the experimental group by Western blotting. The results showed that dectin-1 was expressed at higher levels in cells after LNT treatment than after LPS stimulation (*P* < 0.01) (Figure 3(a)). To further evaluate the anti-inflammatory mechanism of LNT, dectin-1 was blocked by its antagonist Lam. We first evaluated the cytotoxic effect of Lam on BV2 cells. The results showed that Lam had no cytotoxicity to BV2 cells at concentrations of 1-400 *μ*g/ml (Figure 3(b)). In this study, we selected a concentration of 400 *μ*g/ml Lam to block dectin-1 receptor. As shown in Figures 3(c)–3(i), our results indicated that blocking dectin-1 could inhibit the downregulation of Iba1 (*P* < 0.05) (Figure 3(c)), iNOS (*P* < 0.001) (Figure 3(d)), TNF-*α* (*P* < 0.001) (Figure 3(f)), and IL-1*β* (*P* < 0.001) (Figure 3(g)) and the upregulation of Arg-1 (*P* < 0.05) (Figure 3(e)), IL-10 (*P* < 0.05) (Figure 3(h)), and BDNF (*P* < 0.001) (Figure 3(i)) in LPS-induced BV2 cells after LNT treatment. These results suggested that LNT exhibited anti-inflammatory effects in BV2 cells by regulating the dectin-1 receptor.

### 3.4. LNT Ameliorated CPZ-Induced Motor Dysfunction and Demyelination in the Corpus Callosum

**Figure 4:**
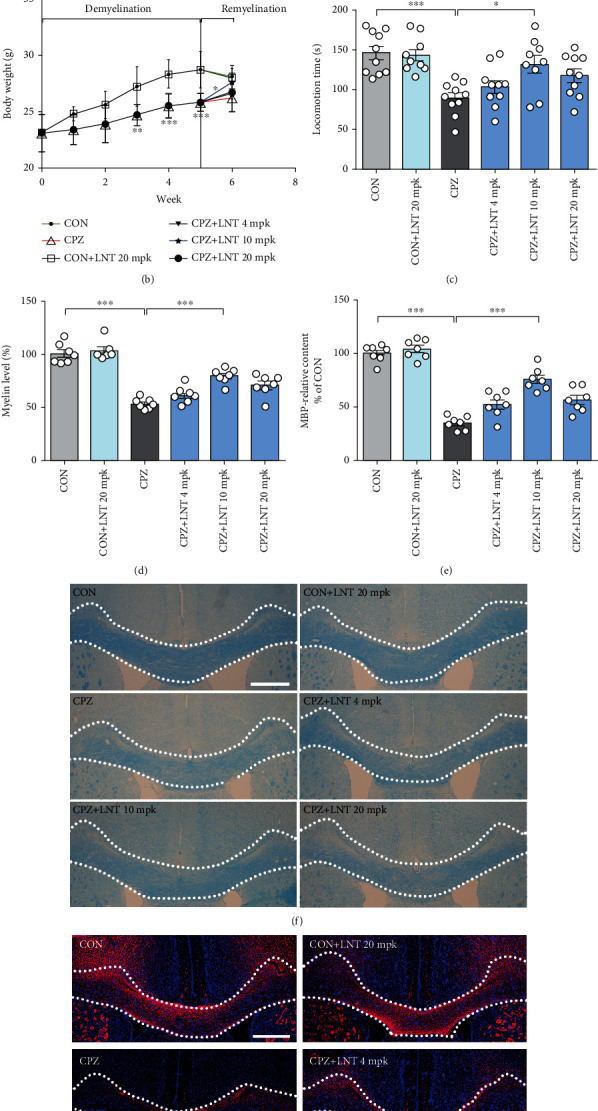
LNT ameliorated CPZ-induced motor dysfunction and demyelination in the CPZ model. (a) Scheme of the experimental protocol of LNT treatment. (b) Effects of LNT on the body weight in CPZ model. (c) Rotarod test. The latency time of each group to stay on the rotarod was recorded. (d) A histogram of quantitative data of the LFB-positive area in the corpus callosum. (e) A histogram of quantitative data of MBP immunostaining in the corpus callosum. (f) Representative images of LFB staining in the corpus callosum. (g) Immunostaining of MBP in the corpus callosum. All data are presented as the mean ± SEM. Scale bar = 200 *μ*m (f, g). Statistical analysis was performed using one-way ANOVA followed by the post hoc Tukey tests. (^∗^*P* < 0.05, ^∗∗^*P* < 0.01, and ^∗∗∗^*P* < 0.001.)

Feeding of CPZ treatment for 5 weeks induces subsequent demyelination [18]. To test the protective effect of LNT in this model, LNT intervention started at the sixth week. The experimental design is shown in Figure 4(a). Compared with the control group (27.20 ± 0.55 g), the CPZ treatment group (24.70 ± 0.30 g) exhibited a significant decrease in body weight in the third week (*P* < 0.01). The most significant weight loss occurred in the CPZ treatment group at weeks four (28.30 ± 0.40 g for CON and 25.50 ± 0.34 g for CPZ) (*P* < 0.001) and five (28.70 ± 0.51 g for CON and 25.80 ± 0.25 g for CPZ) (*P* < 0.001). Compared with the CPZ model group (26.20 ± 0.39 g), LNT treatment (27.60 ± 0.48 g) alleviated body weight loss in the 10 mg/kg group (*P* < 0.05) (Figure 4(b)). The rotarod test was employed to check the improvement of behavior disorders with LNT. Motor coordination ability is shown in Figure 4(c). The locomotion time of CPZ model group mice (89.60 ± 6.61 s) (*P* < 0.001) was significantly decreased compared to the control group (146.40 ± 8.43 s), which was remarkably rescued by LNT at a dose of 10 mg/kg (131.90 ± 11.21 s) (*P* < 0.05). The results above indicated that LNT could significantly ameliorate the motor and coordination impairment caused by CPZ administration. Demyelination was evaluated using LFB staining and MBP staining, which revealed a large area of myelin loss in the corpus callosum after CPZ administration (*P* < 0.001), while LNT intervention reduced the degree of demyelination, especially in the 10 mg/kg treated groups (*P* < 0.001) (Figures 4(d)–4(g)). However, LNT treatment of normal mice showed no effect on motor function or relative myelin expression. Notably, the low dose of 5 mg/kg and the high dose of 10 mg/kg were less effective than the dose of 10 mg/kg, suggesting a dose-dependent effect. Therefore, we used 10 mg/kg as the dose for the experiments to study receptor signaling.

### 3.5. LNT Promoted Remyelination through the Dectin-1 Receptor

**Figure 5:**
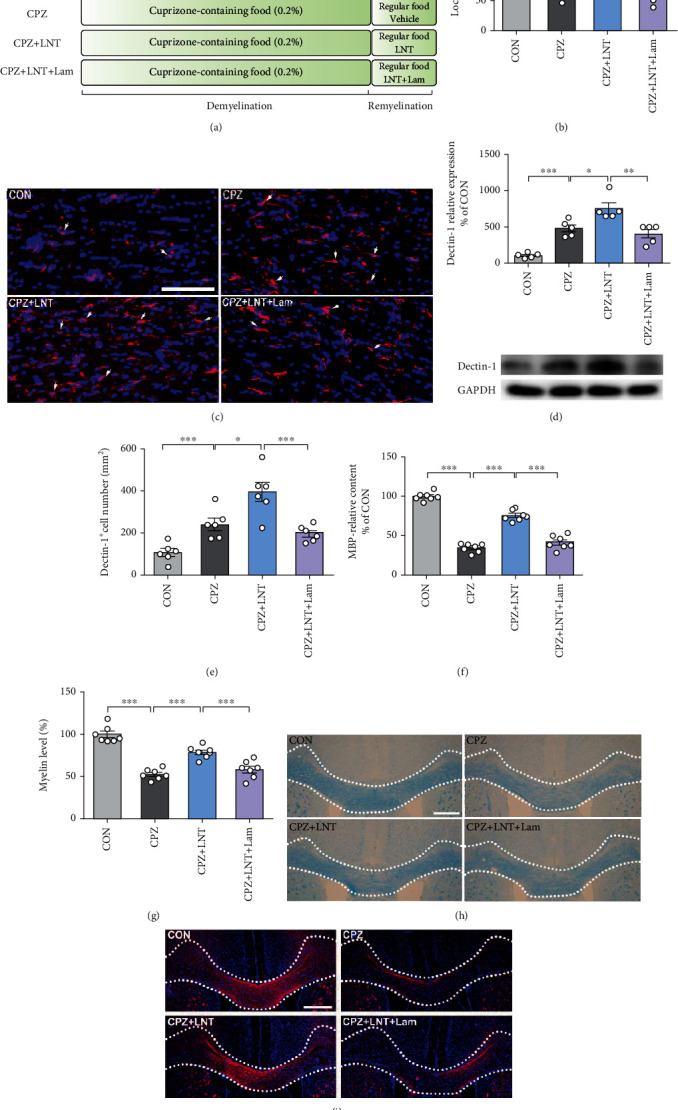
LNT promoted myelin regeneration by regulating the dectin-1 receptor. (a) Scheme of the experimental protocol of LNT and Lam treatment. (b) Rotarod test. The latency time of each group to stay on the rotarod was recorded. (c) Immunostaining of dectin-1 in the corpus callosum. (d) The protein level of dectin-1 was detected by Western blot. (e) Histogram of quantitative data of dectin-1-positive cells. (f) A histogram of quantitative data of MBP immunofluorescence staining in the corpus callosum. (g) A histogram of quantitative data of the LFB-positive area in the corpus callosum. (h) Representative photographs of LFB staining in the corpus callosum. (i) Immunostaining of MBP in the corpus callosum. White arrows indicate representative cells in the treatment group. All data are presented as the mean ± SEM. Scale bar = 100 *μ*m (c); scale bar = 200 *μ*m (h, i). Statistical analysis was performed using one-way ANOVA followed by the post hoc Tukey tests. (^∗^*P* < 0.05, ^∗∗^*P* < 0.01, and ^∗∗∗^*P* < 0.001.)

Previous studies have found that dectin-1 is closely related to the process of regeneration, and *β*-glucan can promote regeneration and repair after optic nerve system injury through the dectin-1 receptor [12]. LNT is a kind of *β*-glucan. To further evaluate the LNT effect on remyelination, dectin-1 was blocked by its antagonist Lam in CPZ model mice, as designed in Figure 5(a). The rotarod test results showed that the effect of LNT on CPZ-induced motor dysfunction was significantly attenuated after dectin-1 was blocked by Lam (*P* < 0.05) (Figure 5(b)). We also examined the expression of dectin-1 in the corpus callosum using Western blotting. We found that the expression of dectin-1 was significantly upregulated after CPZ treatment (*P* < 0.001) and that LNT also upregulated the expression of dectin-1 (*P* < 0.05). Lam treatment significantly downregulated dectin-1 expression in CPZ-treated mice (*P* < 0.01) (Figure 5(d)). Immunofluorescence images showed similar results (Figures 5(c) and 5(e)). LFB staining and MBP immunofluorescence images showed that LNT treatment enhanced myelin content and MBP expression in CPZ-treated mice (*P* < 0.001) but not the antagonist Lam (*P* < 0.001) (Figures 5(f)–5(i)). The beneficial effects of LNT on myelin and MBP levels in the corpus callosum were also significantly inhibited by the dectin-1-specific antagonist Lam.

### 3.6. LNT Regulated Microglial Polarization to Inhibit Neuroinflammation and Promote Remyelination through the Dectin-1 Receptor

**Figure 6:**
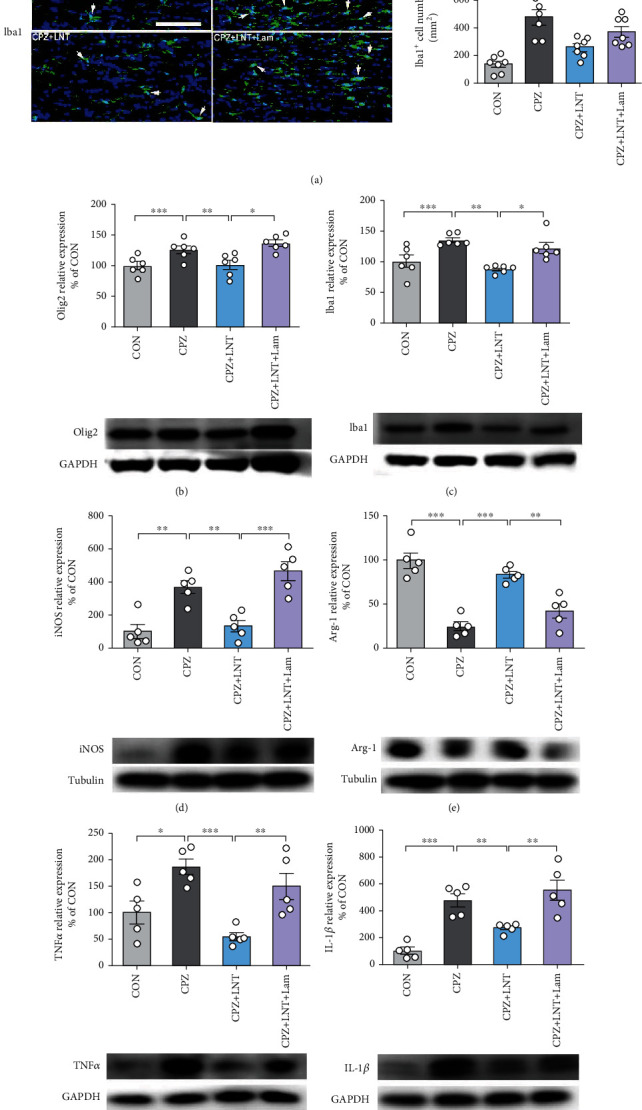
LNT ameliorated CPZ-induced variations in cytokine expression and glial cell activation by regulating the dectin-1 receptor. (a) Representative images and histogram of quantitative data of GFAP-positive cells, Olig2-positive cells, and Iba1-positive cells in the corpus callosum. Histogram of quantitative data of the protein expression levels of Olig2 (b), Iba1 (c), iNOS (d), Arg-1 (e), TNF-*α* (f), IL-1*β* (g), BDNF (h), and IL-10 (i) was detected by Western blot. White arrows indicate representative cells in the treatment group. All data are presented as the mean ± SEM. Scale bar = 100 *μ*m (a). Statistical analysis was performed using one-way ANOVA followed by the post hoc Tukey tests. (^∗^*P* < 0.05, ^∗∗^*P* < 0.01, ^∗∗∗^*P* < 0.001.)

Activation of glial cells is one of the important characteristics of demyelination. Oligodendrocyte precursor cells (OPCs) are recruited to the corpus callosum, which leads to the upregulation of OPCs in the myelin injury area [19–21]. With immunostaining for Olig2, a marker of OPCs, we found that compared with the control group (1331 ± 76 Olig2-positive cells/mm^2^), the number of Olig2-positive cells in CPZ-induced mice (3132 ± 384 Olig2-positive cells/mm^2^) was significantly elevated in the center of the corpus callosum (*P* < 0.001), and LNT treatment (1364 ± 133 Olig2-positive cells/mm^2^) significantly alleviated the CPZ-induced increase in Olig2 cells (*P* < 0.001), but the beneficial effects of LNT were significantly inhibited by the dectin-1 antagonist Lam (1841 ± 99 Olig2-positive cells/mm^2^) (*P* < 0.05) (Figure 6(a)). Demyelination was accompanied by activation of microglia and astrocytes after CPZ treatment. To investigate the effect of LNT on glial cell activation induced by CPZ, we used immunofluorescence to label astrocytes and microglia in the corpus callosum with the marker proteins GFAP and Iba-1, respectively. Compared with the control group (608 ± 71 GFAP-positive cells/mm^2^, 136 ± 23 Iba-1-positive cells/mm^2^), the CPZ group had a significantly increased number of GFAP-positive astrocytes (1129 ± 71 GFAP-positive cells/mm^2^) (*P* < 0.001) and Iba-1-positive microglia (475 ± 52 Iba-1-positive cells/mm^2^) (*P* < 0.001) in the corpus callosum of mice. LNT intervention significantly reversed the increase induced by CPZ administration (768 ± 51 GFAP-positive cells/mm^2^, *P* < 0.05 and 259 ± 26 Iba-1-positive cells/mm^2^, *P* < 0.01). The effect of LNT was blocked when Lam was applied (1017 ± 113 GFAP-positive cells/mm^2^, *P* < 0.05 and 370 ± 40 Iba-1-positive cells/mm^2^, *P* < 0.05) (Figure 6(a)). The same result was also confirmed in Western blot analyses of Olig2 and Iba-1 (Figures 6(b) and 6(c)). Compared with the control group, the CPZ group had a significantly increased Olig2 (*P* < 0.001) and Iba-1 (*P* < 0.001) expression levels in the corpus callosum. LNT intervention reduced the effect on Olig2 (*P* < 0.01) and Iba-1 (*P* < 0.01). Lam intervention significantly reversed the reduce induced by LNT administration (Olig2, *P* < 0.05 and Iba-1 *P* < 0.05).

Western blot analyses were performed to determine the expression levels of proinflammatory and anti-inflammatory cytokines in the corpus callosum of mice. After 7 days of treatment with LNT, compared with the control group, CPZ-treated mice showed significantly increased iNOS (a marker of M1 microglial cells) (*P* < 0.01), TNF-*α* (*P* < 0.05), and IL-1*β* (*P* < 0.001) expression levels in the corpus callosum. However, LNT show obvious inhibitory effect on iNOS (*P* < 0.01), TNF-*α* (*P* < 0.001), and IL-1*β* (*P* < 0.05), while blocking dectin-1 with Lam inhibited the downregulation of proinflammatory factors (iNOS (*P* < 0.001), TNF-*α* (*P* < 0.01), and IL-1*β* (*P* < 0.01)) in the CPZ model (Figures 6(d), 6(f), and 6(g)). Arg-1 (a marker of M2 microglial cells) (*P* < 0.001), BDNF (*P* < 0.05), and IL-10 were downregulated in the CPZ model, which was reversed by LNT treatment (Arg-1 (*P* < 0.001), BDNF (*P* < 0.01), and IL-10 (*P* < 0.01)). However, the effect of LNT was significantly attenuated after dectin-1 was blocked by Lam (Arg-1 (*P* < 0.01), BDNF (*P* < 0.01)) (Figures 6(e), 6(h), and 6(i)).

## 4. Discussion

In the present study, we identified a centrally acting drug, LNT, and when administered in LPS-induced BV2 cells or CPZ animal model, we found that (1) in LPS-treated BV2 cells, LNT promoted the conversion of BV2 cells from M1 status induced by LPS to M2 status; (2) LNT inhibited the proinflammatory cytokines and promoted anti-inflammatory cytokine expression in LPS-induced BV2 cells; (3) the expression of dectin-1 was prominently upregulated, and antagonism of dectin-1 with Lam reversed the effect of LNT; (4) in cuprizone-induced demyelination animal model, LNT significantly enhanced the remyelination and rescued motor deficiency; (5) LNT modulated neuroimmunity to enhance the anti-inflammatory markers IL-10 and BDNF, inhibited inflammatory markers TNF-*α* and IL-1*β*, and downregulated microglia activation and oligodendrocyte and astrocyte proliferation; and (6) antagonism of dectin-1 with Lam abolished LNT-induced demyelination, restoration of motor deficiency, inhibition of Iba1^+^, GFAP^+^, and Olig2^+^ cells, and conversion of microglial cells from M1 status to M2 status. Thus, our study identified LNT as a novel and potential therapeutic agent that can rescue MS neuroimmune dysregulation and reduce demyelination through a dectin-1-dependent mechanism.

Dectin-1, a receptor for *β*-glucans and an immune regulator in the CNS, was identified to mediate the remyelination mechanism of LNT. Dectin-1 receptor signaling is closely related to the neuroimmune system [13]. *Lentinus edodes* is a traditional food, which can improve physiological function, including anti-inflammatory and immunomodulatory. LNT is the main functional component of *Lentinus edodes*. LNT has immune effects and has been reported to bind to *β*-glucan receptors, such as dectin-1, in microglial cells, dendritic cells, monocytes, macrophages, and neutrophils [22, 23]. Dectin-1 recognizes *β*-glucans, a carbohydrate present in the cell walls of many fungal species [24]. It was discovered as the first non-Toll-like receptor capable of coupling Syk-independent pathways, such as those mediated by Raf-1, resulting in the activation of several transcription factors, including NFAT, IRF1, IRF5, and NF-*κ*B [25–28]. Activation of dectin-1 regulates numerous cellular responses, including phagocytosis, autophagy, respiratory bursts, and the production of cytokines [29]. In this study, we further studied the remyelination effect of LNT from the medicinal mushroom *Lentinus edodes* (Berk) Sing. Here, we reported that LNT might bind to its receptor dectin-1 in the brain to exert remyelination effects. Indeed, after LNT treatment in the CPZ-induced demyelination animal model, dectin-1 receptor expression was significantly increased, suggesting that dectin-1 activation was involved in remyelination (Figures 5(c)–5(e)). Lam is a soluble *β*-glucan that is well known to bind dectin-1 with very high affinity [30–35]. A large number of studies have shown that Lam is a typical ligand [36, 37] and a most effective competitive inhibitor of the *β*-glucan receptor dectin-1. There was evidence to show that researcher investigated a set of short *β*-(1→3)-glucans with varying degree of polymerization, 3, 6, 7, 16, and Lam, analyzing the relationship between the structure and interaction with the C-type lectin-like domain (CTLD) of dectin-1. The domain interacted strongly with Lam by systematically analyzing it, plausibly forming oligomeric protein-Lam complexes, and Lam has a secondary structure [38]. Consistent with the previous studies, we found that the remyelination effects of LNT were almost completely blocked by the dectin-1-specific inhibitor Lam (Figures 5(f)–5(i)). These results suggest that targeting dectin-1 may become a novel strategy for remyelination.

Microglia continuously survey the microenvironment with their motile protrusions, making them ready to respond to insults, including viral and bacterial infections, toxins, and local tissue injury [39–41]. Upon activation, microglia lose their homeostatic gene signature and undergo disease-specific changes. Polarization of microglia was originally explained by the M1-M2 dichotomy: classical- (M1-) activated microglia were considered to be deleterious by releasing destructive proinflammatory mediators, whereas alternative (M2) microglia are involved in the resolution of inflammation and phagocytosis [42]. However, previous studies have shown that most compounds can suppress neuroinflammation simply by inhibiting M1 microglial activation [43–45], and few compounds can suppress neuroinflammation by promoting the conversion of M1 microglia to M2 microglia [46–48]. Our results showed that LNT treatment significantly inhibited the upregulation of iNOS and the downregulation of Arg-1 expression (Figures 2(a)–2(c)), which indicates that LNT functions as a molecular switch to convert microglia from the M1 to the M2 type, alleviates inflammation, and promotes remyelination.

Dectin-1 can induce or regulate numerous cellular responses, including phagocytosis, the respiratory burst, neutrophil extracellular trap formation, autophagy, dendritic cell maturation and antigen presentation, inflammasome regulation, and the production of eicosanoids, cytokines, and chemokines [13]. LNT is a *β*-glucan [7]. Previous studies have shown that *β*-glucans can stimulate dectin-1 or TLR2, which leads to the increased permeability of the blood-brain barrier [23, 49]. Here, we reported that LNT might bind to its receptor dectin-1 and modulate microglial polarization in the brain to exert remyelination effects (Figure 5). The dectin-1 receptor levels were significantly increased after CPZ-induced demyelination in the animal model and LNT treatment (Figures 5(c)–5(e)). Dectin-1 inhibition by Lam almost completely abolished the remyelination effects of LNT (Figures 5 and 6). We found that LNT switched the brain from an inflammatory status to a repair and regeneration status via modulation of dectin-1-mediated microglial polarization in the brain. Rescue of the innate immune balance of the proinflammatory status to anti-inflammation and regeneration status would have a considerable impact on the development of new effective treatments for MS for the improvement of remyelination and motor function in the recovery stage. In patients with MS and in models of demyelination, CNS inflammation homoeostasis is destabilized by a variety of mechanisms [50]. Our previous studies showed that both *Ganoderma lucidum* polysaccharides and proteo-*β*-glucan from maitake exert their anti-inflammatory effects through the modulation of dectin-1 [32, 51]. Therefore, this study confirmed that LNT, like other polysaccharides containing *β*-glucan, also mediated the anti-inflammatory effect through the modulation of dectin-1.

In conclusion, this study showed that LNT significantly improved central demyelinating disease by regulating dectin-1, promoting the conversion of microglial cells from the M1 status to the M2 status, enhancing anti-inflammatory and regeneration, inhibiting the occurrence of neuroinflammation, improving motor function and remyelination, and providing a novel potential candidate medicine for the remyelination stage in the treatment of central demyelination-associated diseases.

## Data Availability

The data used to support the findings of this study are included within the article.
